# Sleep problems are a strong predictor of stress-related metabolic changes in police officers. A prospective study

**DOI:** 10.1371/journal.pone.0224259

**Published:** 2019-10-22

**Authors:** Sergio Garbarino, Nicola Magnavita

**Affiliations:** 1 Post-graduate School of Occupational Health, Università Cattolica del Sacro Cuore, Rome, Italy; 2 State Police Health Service Department, Ministry of the Interior, Rome, Italy; 3 Department of Neuroscience, Rehabilitation, Ophthalmology, Genetics and Maternal-Infantile Sciences (DINOGMI), Genoa, Italy; 4 Department of Woman/Child & Public Health, Fondazione Policlinico Gemelli IRCCS, Rome, Italy; Instituto Mexicano del Seguro Social (IMSS) HGZ 2, MEXICO

## Abstract

**Objective:**

Previous studies have shown that workers chronically exposed to occupational stress have an increased risk of metabolic syndrome (MetS) and sleep problems (SPs). The purpose of this study was to verify whether SPs mediate the relationship between stress and MetS.

**Method:**

A 5-year prospective cohort study included 242 police officers from a rapid response unit engaged exclusively in maintaining law and order. Perceived stress levels were measured repeatedly with the demand-control-support and the effort-reward-imbalance questionnaires; insomnia symptoms were assessed with the Pittsburgh Sleep Quality Index; excessive daytime sleepiness was measured using the Epworth Sleepiness Scale. MetS and its components were evaluated at baseline and at follow-up.

**Results:**

During 5-year follow-up period, 26 new cases of MetS were identified. Both occupational stress and SPs were significantly related to incident cases of MetS. Insomnia symptoms showed a highly significant association with MetS (aOR 11.038; CI95% 2.867–42.493). Mediation analysis confirmed that SPs mediate the relationship between stress and MetS.

A reciprocal relationship was found between job stress and SPs. Work-related stress was a significant predictor of insomnia symptoms, short sleep duration, sleep dissatisfaction, and sleepiness. Compared to the reference group, police officers with SPs at baseline had significantly higher odds of reporting high stress at follow-up.

**Conclusion:**

SPs play a mediating role in the relationship between occupational stress and MetS. Prevention of MetS must include the control of stress factors and an increase in the resilience of workers, but correct sleep hygiene is also an essential factor.

## Introduction

Metabolic syndrome (MetS) is a cluster of metabolically related cardiovascular risk factors (obesity, insulin resistance, dyslipidaemia and hypertension) [1] that also predict a high risk of developing diabetes [2], cancer [3], and cognitive impairment [4]. MetS is highly prevalent and increasing in most parts of the world [5].

Chronic occupational stress is considered to be a risk factor for MetS [6–16]. A recent meta-analysis of longitudinal studies showed that work-related stress is associated with a significant increase in the pooled risk of metabolic syndrome (RR = 1.47; CI95% = 1.22–1.78) [17]. In a previous longitudinal study on a rapid response police unit we observed that police officers with high job strain had a higher adjusted risk of developing MetS (aOR = 2.68; CI95% = 1.08–6.70) and hypertriglyceridemia (aOR = 7.86; CI95% = 1.29–48.04) than their colleagues classified in the lowest quartile of work-related distress [18].

It is well known that sleep disorders, such as obstructive sleep apnea (OSAS), are associated with MetS [19, 20]. It would be interesting to determine whether alterations in the quantity and quality of sleep that can occur in working environments, regardless of the existence of morbid conditions such as OSAS, are able, if continued over time, to induce MetS. Studies that consider only sleep duration have produced inconsistent results. For example, short sleep duration proved to be an independent risk factor for MetS in longitudinal studies conducted in Korea [21], China [22], Canada [23] and Japan [24], while in an Iranian cohort study, long sleep duration increased the risk of MetS [25]. In a Swedish cohort, both short and long sleep durations, and problems with falling and staying asleep were linked to a higher prevalence of MetS [26]. In a sample of police officers, poor sleeping conditions, i.e. lack of sound sleep and shift sleep disorder, were associated with a high prevalence of MetS [27]. A recent review of existing cross-sectional studies [28] provided some evidence of a positive association between sleep quality, or some sleep complaints (difficulty in falling asleep, difficulty in maintaining sleep, and sleep inefficiency) and MetS. However, further studies based on a longitudinal design are needed to explore the causal relationship between sleep quality and MetS.

Work is known to disturb sleep, due to occupational exposure to many stressors [29]. Some aspects of work organization, such as lack of control over work times [30], insufficient rest between shifts [31], psychosocial burden and night work [32] or shift work [33] are predictors of sleep problems. Exposure to workplace violence is also associated with a significant increase in the pooled risk of sleep problems [34]. More generally, there is evidence that psychosocial stress is associated with sleep problems [35], and that both sleep deprivation [36, 37] and low sleep quality [38–40] are associated with stress response. Due to considerable disparity in the results of existing studies, the degree to which the relationship between job stress and sleep symptoms is unidirectional or reciprocal is unclear.

The questions to be answered in this study are: can the presence of sleep symptoms be a predictor of MetS, and in what way are sleep, stress and metabolic syndrome related? To answer these questions, we used the same cohort of workers whose surveillance revealed the existence of an association between stress and metabolic syndrome. In this cohort we studied the evolution of sleep symptoms. The main aim of the research was to assess the extent to which sleep problems were associated with an increased risk of MetS at the end of a five–year period of observation. The collateral objective was to study the relationships between occupational stress and sleep problems.

## Methods

### Subjects and ethics statement

The study population, which was described in detail in the previous study [18] was composed of police officers from the “VI Reparto Mobile” of Genoa. This rapid response police unit was chosen because it is exclusively engaged in maintaining law and order and is therefore homogeneously exposed to relevant factors of occupational stress. In this paper, in accordance with the literature [41–43], we used the term "stress" to describe workplace stressors that lead to strain [44], imbalance [45] or other negative responses or reactions, called “distress”. Studies on this cohort showed that occupational stress can be higher during routine, but often unpredictable activities, than during highly important public events when activities are carefully programmed in advance [46]. Although occupational exposure was homogeneous, as all the police officers performed the same tasks, personal response to stressors varied due to difference in personality [47] or individual characteristics [48]. As a result, the health consequences of stress were diverse [49]. The study population was not exposed to some of the confounding factors of MetS, such as a sedentary lifestyle, food or alcohol abuse or drug use since all the police officers regularly followed an intensive weekly program of physical exercise and a healthy diet, designed specifically to guarantee a correct nutritional intake. At the time of enrolment, the police officers were in good health and had no sleep-disordered breathing or other diseases associated with MetS.

In 2009, prior to an international G8 meeting in which they were responsible for maintaining law and order, the police officers were invited to participate in a work-related health program that included frequent monitoring. The study plan had previously been approved by the Italian Ministry of the Interior and by all police trade-union organizations. An information leaflet informed participants of the voluntary and confidential nature of the study through an information leaflet. All but two police officers accepted (292 out of 294 police officers) and gave their written informed consent by signing a form in their personal health file report. Written informed consent for access to health records and linkage was also obtained in compliance with indications in the literature [50] and the Italian Law on Privacy [51]. The study was conducted according to the ethical standards of the Declaration of Helsinki. Two female participants were excluded from subsequent analyses in order to avoid gender bias. At the end of the observation period, the cohort was composed of 234 police officers. No worker dropped out of the study, and no police officer ever submitted incomplete or non-assessable data. The reasons for abandoning the cohort were: transfer to other police units (41 persons, 73.2%); retirement (12, 21.4%), and death (3, 5.4%).

### Questionnaires

Data were collected from January 2009 (T1) to January 2014 (T2) through medical examination and questionnaires. Self-assessment of job stress was performed during the 5-year period of observation by repeatedly completing the Italian version [52] of Karasek’s demand-control-support (DCS) questionnaire [44], and Siegrist’s Effort–Reward Imbalance (ERI) questionnaire [45]. In this cohort Cronbach’s α values for the sub-scales of DCS, job demand and job control, were 0.71 and 0.65, i.e. close to those found in the literature [53–57], thus indicating reasonably acceptable internal consistency [58]. The Cronbach’s α values for the sub-scales job effort and job reward of the ERI questionnaire were 0.82 and 0.89, respectively, showing high internal consistency. Blood tests and medical examinations were performed at baseline (T1) and at the follow-up (T2).

In addition to the aforementioned and already published data, in 2009 and 2014, the police officers also answered some questions from the Pittsburgh Sleep Quality Index (PSQI), [59] a self-report measure of sleep quality over a 1-month time interval, and from the SDS (sleep disorder score), an Italian questionnaire on sleep [60], in compliance with a validated protocol [61]. Evidence indicates that the PSQI is a highly reliable and valid questionnaire [62]. In this study the selected items had a Cronbach’s alpha of 0.717. The questionnaire provided a numerical variable (HS, hours of sleep, in response to the question: “During the past month, how many hours of actual sleep did you get at night?”) and three ordinal variables (DS, difficulty sleeping; SI, sleep interruptions; EA, early awakenings) from a frequency Likert scale score of 1 to 4 in response to the following questions: “During the past month, how often have you had trouble falling asleep within 30 minutes?”, “During the past month, how often have you had sleeping problems because you wake up in the middle of the night?”, “During the past month, how often have you had sleeping problems because you wake up early in the morning?”. HS were also recoded in a binary variable: police officers who slept less than 7 hours were classified as insufficient sleepers. The duration of daily sleep was then used both as an outcome (with two categories) and as a predictor variable.

The ranked T1 and T2 scales (DS, SI and EA) were also divided into no or infrequent symptoms (not during the past month, less than once a week, or once a week) and experiencing symptoms (more than once a week). The variables were used both as an outcome (with two categories, divided as described above) and as a predictor. Moreover, since insomnia is defined in the International Classification of Sleep Disorders, Third Edition [63] as difficulty in initiating or maintaining sleep, while the Australasian Sleep Edition indicates that it is a disorder characterized by difficulty with sleep initiation, sleep maintenance, early awakening, or a combination of the above difficulties [64], and the DSM-5 categorizes insomnia disorder as a sleep-wake disorder, characterized by dissatisfaction with sleep quantity or quality, associated with one (or more) of the aforementioned symptoms [65], we pooled the DS, SI and EA ranked scales in a single variable that measured insomnia symptoms. A principal component analysis of the aforementioned ordinal variables was conducted to ensure that sleep disorder variables could be incorporated in a single variable. The results suggested a one-factor solution (R^2^ = 44.22% of the variance with T1 variables, R^2^ = 50.96% of the variance with T2 variables). The insomnia symptom scale (IS), that had values ranging from 3 to 12, was used as a predictor variable.

Another ordinal variable, sleep satisfaction (SS), was obtained from a 4-point Likert scale response to the question: “During the past month, how satisfied were you with your overall sleep quantity and quality?” This variable was also used both as an outcome (with two categories) and as a predictor variable.

Sleepiness was evaluated using the Epworth Sleepiness Scale ESS [66], a self-administered questionnaire with 8 questions that rated on a 4-point scale (0–3) the chances of dozing off or falling asleep while engaged in eight different activities. A systematic review has shown that the internal consistency of the questionnaire measured by Cronbach’s alpha is good [67]. In our sample the alpha value (0.79) fell within the limits of the literature (0.73–0.86). ESS score was used as a predictor. An ESS score >10 indicated excessive daytime sleepiness (EDS) [68,69]; EDS was used as a binary outcome.

Both the demand / control (D/C) ratio and the effort / reward (E/R) ratio were used to assess occupational stress level. Karasek’s and Siegrist’s stress models are complementary and, if used together, make it possible to evaluate both the effect of the interaction between workloads and control, and the transactional effects caused by disparity between effort and the results obtained. As is known, there is no safe level: the effects of stress depend on individual resources and on the perception that each individual has of his/her own condition. Since police officers were exposed to homogeneous conditions of work stress, our aim was to determine the relative risk that each individual attributed to this exposure. For each of the five measurements made during the period, the quartiles of the two distributions were added and the final result was again subdivided into quartiles. In this way, the population was divided into four groups; the first consisted of subjects who reported the highest levels of stress, whereas the fourth was composed of resilient police officers who, although performing identical tasks, perceived the lowest levels of stress. By grouping the first two categories and the last two, a binary variable was also constructed, consisting of a high and low level of perceived stress.

The International Diabetes Federation (IDF) guide [70] and the National Cholesterol Education Program Expert Panel on Detection Evaluation and Treatment of High Cholesterol in Adults (NCEP/ATPIII) [71] were used to define metabolic syndrome components as follows: central obesity was considered to be a BMI>30 kg/m^2^, or a waist circumference of >85 cm in men. Hypertriglyceridemia was defined as a serum triglyceride level >150 mg/dL (1.7 mmol/L), while low high-density lipoprotein (HDL) cholesterolemia was represented by a serum HDL-cholesterol level of <40 mg/dL (1.03 mmol/L). High blood pressure was defined as a systolic blood pressure of >130 mmHg and/or a diastolic blood pressure of >85 mmHg. High fasting glucose was defined by a plasma glucose level of >100 mg/dL (5.6 mmol/L).

### Statistical analyses

Firstly, the variables of interest were analyzed by assessing central tendency and variability measures. Comparison of the values of stress-related variables was made by Student’s t-test for coupled data, and the prevalence of sleep problems at baseline and at follow-up was compared by means of the chi-square test. The prevalence and incidence of metabolic disorders were also assessed.

In a further step, a study was made of the relationship between stress and sleep problems. The four groups classified according to the level of stress were compared by one-way analysis of variance (Anova) and the Bonferroni post-hoc comparison of group means to ascertain how sleep problems were distributed at the beginning and end of the observations. The distribution of sleep hours (SH); insomnia symptoms (IS) including difficulty sleeping (DS), sleep interruptions (SI) and early awakenings (EA); sleep satisfaction (SS) and daytime sleepiness (Epworth scale score, ESS) were analyzed in relation to stress levels. In this way we were able to ascertain whether, within the population, those who perceived a higher level of occupational stress also had a higher frequency of sleep problems. Hierarchical logistic regression analyses were then performed, in order to evaluate the unidirectional or bidirectional path of the association. The variables for job stress or sleep problems were entered in the first set to estimate the crude odds ratios. Subsequently, demographic variables [i.e. age (years), education level (dichotomized at 8 years of schooling), rank (officer or superior/technician), geographic origin (Northern or Southern Italy), housing (in barracks or home), marital status (single or divorced/ married or cohabiting) and presence of offspring (no/yes)] were added. The baseline variable of sleep or stress (depending on the research hypothesis) was included in the third set. The remaining covariates were added in the final set.

As a third step, multiple hierarchical logistic regression analysis was used to ascertain whether sleep problems or a combination of sleep problems and job stress were associated with metabolic syndrome incidence. Initially (model I), the average number of hours slept per day was entered as a predictor, and the incidence of metabolic syndrome as a dependent variable. In the following models, daytime sleepiness (model II), work-related stress (model III), and insomnia symptoms (model IV) were entered as predictors. Analyses were adjusted for confounding factors (age, rank, education, geographic origin, marital status, housing, offspring).

Lastly, univariate and multivariate logistic regression analyses were used once more to determine which of the sleep problems best correlated with the incidence of MetS and its components.

An assessment of the possible mediation effect of insomnia in the causal pathway linking occupational stress and metabolic syndrome was performed using the approach proposed by Hayes [72]. Firstly, calculations were made of the total (unadjusted) effect of occupational stress on metabolic syndrome as well as the regression coefficients of the links between occupational stress and insomnia (“a” pathway—by linear regression analysis) and between insomnia and metabolic syndrome (“b” pathway—by logistic regression analysis). Secondly, a multiple logistic regression model was constructed by taking metabolic syndrome as the dependent variable and both occupational stress and insomnia (i.e. the mean value of insomnia scores at baseline and at the end of follow-up) as independent variables. Lastly, the bootstrapping indirect effect of occupational stress (i.e. mediated by insomnia) was calculated and expressed by regression coefficient and bootstrapping confidence interval. The number of bootstrap samples was fixed at 5000. Analyses were performed using the Statistical Package for Social Science (IBM/ SPSS) for Windows (rel. 23.0).

## Results

The characteristics of the observed sample are reported in Table 1. At baseline, the mean age was 36.0+7.4 years, and the average length of work experience was 14.6+7.8 years. According to Italian educational standards, three out of four of the participants had an average or high level of education (>8 years of education). Rank was basic (officer) for approximately half of the sample, while it was higher (special officer, superintendent, inspector, technician) for the other half, with only slight differences in salary between the various ranks, and practically no difference in the tasks performed. Half of the participants were born in Northern Italy, while the remainder came from Southern Italy. Most of the participants were quartered in barracks.

**Table 1:** Characteristics of the population studied.

Social-demographic & lifestyle variables			
Gender, male N (%)	234 (100%)		
Age, years (mean ± s.d.)	36.0 ± 7.4		
Working experience, years (mean ± s.d.)	14.6 ± 7.8		
Rank, superior to officer, N (%)	126 (53.8%)		
Education level, >8 years, N (%)	170 (72.6%)		
Origin, Northern Italy, N (%)	120 (51.3%)		
Marital status, married, N (%)	93 (39.7%)		
Housing, barracks, N (%)	120 (51.3%)		
Offspring, presence, N (%)	94 (40.2%)		
Smokers, N (%)	66 (28.2%)		
Job stress variables			
	Baseline	Follow-up	*p*^a^
Demand (range 5–20) (mean ± s.d.)	14.18±1.88	14.18±1.94	n.s.
Control (range 6–24) (mean ± s.d.)	11.77±2.49	11.89±2.56	<0.01
Support (range 6–24) (mean ± s.d.)	17.76±2.92	17.80±2.91	n.s.
Effort (range 6–30) (mean ± s.d.)	17.22±3.10	17.34±3.16	<0.01
Reward (range 11–55) (mean + s.d.)	37.89±5.57	38.02±5.48	<0.05
Over-commitment (range 6–24) (mean ± s.d.)	7.12±2.21	7.42±2.28	<0.001
Job strain (D/C weighted ratio)	1.52±0.44	1.51±0.45	n.s.
Effort/Reward ratio	0.87±0.31	0.87±0.31	n.s.
Sleep duration			
	Baseline	Follow-up	*p*^a^
Hours slept, on average (mean + s.d.)	6.7 ± 1.1	6.4 + 1.2	<0.001
Prevalence of sleep problems			
	Baseline	Follow-up	*p*^b^
Insufficient sleep time (<7hours), N (%)	109 (46.6)	123 (52.6)	<0.001
Difficulty Sleeping, (>1/wk) N (%)	7 (3.0)	21 (9.0)	n.s.
Sleep Interruptions, (>1/wk) N (%)	12 (5.1)	26 (11.1)	<0.001
Early Awakenings, (>1/wk) N (%)	12 (5.1)	17 (7.3)	<0.001
Sleep dissatisfaction, N (%)	56 (23.9)	64 (27.4)	<0.001
Sleep quality problems, N (%)	69 (29.5)	86 (36.8)	<0.001
Excessive Daytime Sleepiness, N (%)	42 (17.9)	67 (28.6)	<0.001
Prevalence of metabolic disorders			
	Baseline	Follow-up	*p*^b^
Abdominal obesity (N, %)	74 (31.6)	186 (79.5)	<0.001
Hypertension (N, %)	32 (13.7)	73 (31.2)	<0.001
Hypertriglyceridemia (N, %)	34 (14.5)	42 (17.9)	<0.001
Low HDL-cholesterol (N, %)	26 (11.1)	37 (15.8)	<0.001
Hyperglicaemia (N, %)	7 (3.0)	10 (4.3)	<0.001
Metabolic syndrome (N, %)	14 (6.0)	40 (17.1)	<0.001

^(a)^: Student’s t-test

^(b)^: Chi-square test, Fisher’s exact test

Stress levels showed some fluctuations during the observation period. A comparison of average follow-up and baseline values revealed a significant increase in control, effort, reward, and over-commitment. However, no significant changes were observed in composite distress measures, i.e. D/C and E/R weighted ratios (Table 1). During the 5-year observation period, 207 police officers (88.5%) reported a mean D/C ratio>1, which is considered a condition of job strain, and 29 (12.4%) reported a mean E/R ratio>1, considered to be indicative of effort/reward imbalance. An analysis of the different measurements performed during the 5-year period indicated that 45 officers (19.2) had high stress levels, 31 (13.2%), 82 (35%) were classified in the intermediate quartiles of distress, and 76 (32.5%) manifested low job-related stress.

The initial overall sleep hygiene picture was not optimal, with about half of the workers reporting insufficient sleep (sleeping less than 7 hours a day) and one in four claiming to be dissatisfied with the quantity or quality of sleep. At follow-up, all sleep indicators had worsened. On average, sleep time declined from 6.7 ± 1.1 to 6.4 ± 1.2 hours (t Student’s t for paired data = 6.29; *p* <0.001). The share of those presenting DS, SI, or EA increased from 12% to 21%; however, pre-post statistically significant differences were found only for sleep interruptions (5.1% vs. 11.1%, *p*<0.001), early awakening (5.1% vs. 7.3%, *p*<0.001), sleep dissatisfaction (23.9% vs. 27.4%, *p*<0.001) and daytime sleepiness (17.9% vs. 28.6%, *p*<0.001). The prevalence of sleep quality problems (DS, SI, EA, SS) increased from 29.5% at T_1_ to 36.8% at T_2_ (*p*<0.001) (Table 1).

All the components of metabolic syndrome were found to have a significantly higher prevalence at follow-up than at baseline (chi square or Fisher’s exact test *p*<0.001). Over the 5-year period 112 new cases of abdominal obesity (incidence:0.7), 41 of hypertension (0.20), 8 of hypertriglyceridemia (0.04), 11 of low HDL-cholesterol (0.05), and 3 of hyperglycemia (0.01) were observed leading to the diagnosis of 26 new cases of metabolic syndrome (0.12).

One-way Anova demonstrated that sleep symptoms differed significantly between groups of officers characterized by different levels of stress (*p*<0.001). Post-hoc comparisons using the Bonferroni test confirmed that the police officers with persistently lower levels of perceived stress had significantly better sleep conditions than their colleagues in the highest quartile of job strain. The police officers in the lowest stress quartile were significantly more satisfied with the quality and quantity of sleep, and had fewer insomnia symptoms and lower daytime sleepiness than other groups (*p*<0.001 for all comparisons). On average, the non-distressed police officers slept more than 7 hours a day (on average, 7.1±0.9 hours), while the policemen in the highest quartile of perceived job stress slept less than 6 hours a day (5.0±0.8; Student’s t test *p*<0.001) (Table 2).

**Table 2:** Stress and sleep problems. Difference in prevalence of sleep complaints (average scores) between groups exposed to different levels of work stress at baseline and at follow-up.

	Stress level				ANOVA F	*p*
	Lowest quartile	2^nd^ quartile	3^rd^ quartile	Highest quartile		
*Baseline*						
IS	4.9±1.1	5.7±1.8	6.2±1.6	6.9±1.7	18.06	<0.001
SS	3.6±0.7	3.5±0.7	2.8±0.7	2.3±0.7	47.14	<0.001
SH	7.2±0.9	6.9±0.9	6.2±0.7	5.6±0.7	41.05	<0.001
ESS	4.4±3.3	5.0±3.7	6.9±2.8	9.2±2.3	24.41	<0.001
*Follow-up*						
IS	5.1±1.1	6.2±1.7	7.2±1.7	8.6±1.8	51.39	<0.001
SS	3.7±0.6	3.4±0.7	2.5±0.7	2.0±0.7	64.05	<0.001
SH	7.1±0.9	6.7±0.9	6.0±0.8	5.0±0.8	55.38	<0.001
ESS	4.5±3.0	5.9±3.9	8.7±3.3	11.0±2.7	41.86	<0.001

IS, insomnia symptoms (difficulty sleeping; sleep interruptions; early awakenings); SS sleep satisfaction; SH sleep hours; ESS Epworth scale for daytime sleepiness

**Fig 1:**
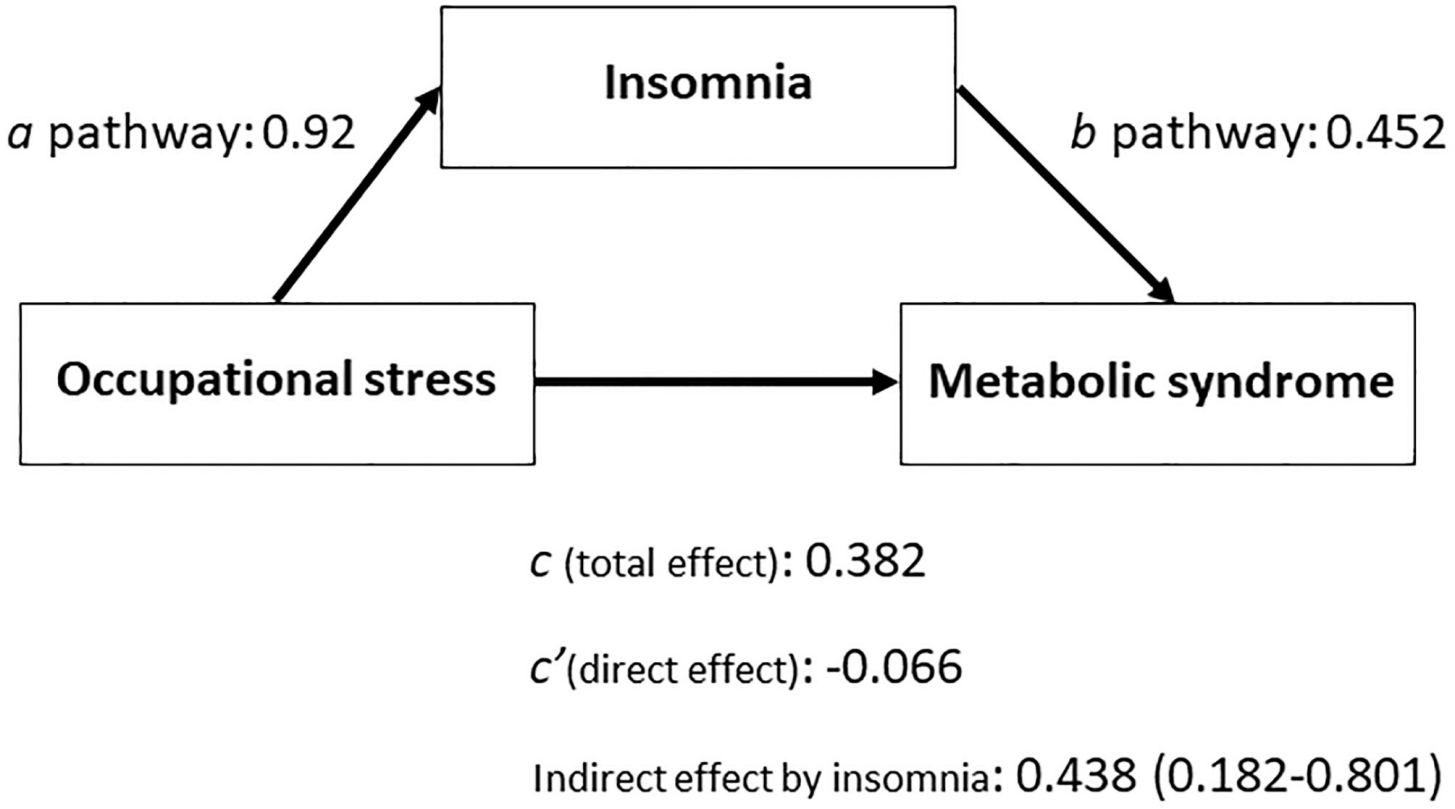
Mediation analysis of the inter-relationships between occupational stress, insomnia and metabolic syndrome (unstandardized coefficients).

Sleep problems were significantly related to incident cases of metabolic syndrome. The average number of hours of sleep had a protective effect. Using logistic regression analysis, the association between the number of hours slept and the prevalence of MetS was non-significant at baseline (OR 0.736; CI95% 0.434–1.247). At follow-up sleep hours were significantly associated with MetS prevalence (OR 0.568; CI95% 0.414–0.780; *p*<0.001). In longitudinal observation the average number of hours slept during the observation period was significantly associated with the occurrence of MetS cases (crude OR 0.476; CI95% 0.310–0.730; *p*<0.001). The adjusted OR (corrected for age, rank level, education, geographic origin, marital status, housing, and presence of offspring) was 0.452 (CI95% = 0.289–0.710; *p* = 0.001), and remained significant even after correction for sleepiness, stress and sleep satisfaction (aOR 0.468; CI95% = 0.2327–0.942; *p* = 0.033). However, a much stronger association was observed between the quality of sleep and MetS than between the latter and the quantity of sleep: after adding insomnia symptoms to the multivariate hierarchical logistic model, hours of sleep were no longer significant, and insomnia symptoms showed a highly significant aOR (11.038; CI95% = 2.867–42.493; *p*<0.001). The coefficient of determination of the model improved significantly when insomnia symptoms were introduced into the analysis (Table 3) (Fig 1).

**Table 3:** Association of sleep problems with incident cases of metabolic syndrome. Hierarchical regression analysis.

Variable	Adjusted*, Model 1			Model 2**			Model 3***			Model 4****			Model 5****			
	OR	95% CI	*p*	OR	95% CI	*p*	OR	95% CI	*p*	OR	95% CI	*p*	OR	95% CI	*p*
Sleep Hours^#^	0.452	0.289–0.707	.000	0.422	0.240–0.743	.003	0.403	0.210–0.776	.007	0.468	0.232–0.942	.033	0.481	0.230–1.006	n.s.
ESS score^#^				0.972	0.844–1.119	n.s.	0.980	0.841–1.140	n.s.	0.966	0.827–1.129	n.s.	0.912	0.769–1.083	n.s.
Stress							0.921	0.510–1.661	n.s.	0.830	0.446–1.547	n.s.	0.746	0.381–1.463	n.s.
Sleep satisfaction^#^										0.669	0.330–1.355	n.s.	0.776	0.370–1.626	n.s.
Insomnia symptoms^#^													11.038	2.867–42.493	.000
Nagelkerke R^2^	.175			.176			.177			.186			.284		

^#^ average T_1_ and T_2_ measurements

*corrected for age, rank level, education, geographic origin, marital status, housing, and presence of offspring

** also corrected for excessive daytime sleepiness

***also corrected for stress

****also corrected for sleep satisfaction

Findings for the relationship between insomnia symptoms and MetS components revealed an unadjusted significant association with incident cases of dyslipidemia (hypertriglyceridemia OR = 62.65; CI95% 9.33–420.72; *p*<0.001; low HDL-cholesterol OR = 9.96; CI95% 2.28–43.54; *p* = 0.002). The effects of insomnia symptoms were robust as the addition of adjustment variables in three separate sets failed to produce noticeable alterations in these estimates (Table 4).

**Table 4:** Metabolic effects of insomnia. Odds ratios and 95% confidence intervals of Insomnia symptoms (DS, SI, EA) on incident cases of metabolic syndrome and its components. Hierarchical regression analysis.

Output (exposed cases, N)	Unadjusted			Adjusted, Model 1^a^			Adjusted, Model 2^b^		
	OR	95% CI	*p*	OR	95% CI	*p*	OR	95% CI	*p*
MetS (220)	12.91	4.43–37.60	.000	14.17	4.58–43.84	.000	11.04	2.87–42.49	.000
Abdominal obesity (160)	2.11	0.64–6.90	n.s.	2.94	0.84–10.27	n.s.	5.83	1.34–25.45	.019
Hypertension (202)	1.29	0.44–3.83	n.s.	1.39	0.45–4.22	n.s.	2.89	0.76–11.07	n.s.
Hypertriglyceridemia (200)	62.65	9.33–420.72	.000	108.93	10.01–1178.34	.000	n.e.	n.e.	n.e.
Low HDL-cholesterol (208)	9.96	2.28–43.54	.002	13.01	2.41–70.12	.003	6.97	1.06–45.99	.044
Hyperglicaemia (227)	0.97	0.03–35.16	n.s.	1.30	0.03–52.38	n.s.	0.46	0.01–69.91	n.s.

^(a)^ corrected for age, rank level, education, geographic origin, marital status, housing, presence of offspring

^(b)^ also corrected for average hours of sleep, ESS score, sleep satisfaction and stress

n.s.: not significant

n.e.: not evaluable

The crude estimates showed that high stress among policemen predicted higher odds of sleep disorders at follow-up compared to the reference group. Work-related stress was a significant predictor of insomnia symptoms (Table 5), short sleep duration (Table 6), sleep dissatisfaction (Table 7), and sleepiness (Table 8). Adjusted ratios were still significant, although the associations were weaker.

**Table 5:** Effect of stress on insomnia symptoms. Multivariate hierarchical logistic regression analysis.

Stress group	Model I^a^		Model II^b^		Model III^c^		Model IV^d^	
	OR	CI95	OR	CI95	OR	CI95	OR	CI95
High stress	30.42***	8.33–111.13	45.37***	11.18–184.10	31.03***	6.95–138.53	11.41*	1.80–72.15
Intermediate high	9.96***	2.48–40.00	10.23***	2.42–43.37	6.40*	1.34–30.50	3.70	0.67–20.34
Intermediate low	4.17*	1.30–15.41	4.92*	1.26–19.33	3.45	0.78–15.24	3.22	0.70–14.78
Low stress	Ref.		Ref.		Ref.		Ref.	

Notes.

*** p < .001,

** p < .01,

* p < .05.

CI95 = 95% confidence interval.

^a^ Crude estimates.

^b^ Adjusted for age, education, rank level, geographic origin, marital status, housing, and presence of offspring.

^c^ Adjusted for Model 2 + insomnia symptoms at T1.

^d^ Adjusted for Model 3 + excessive daytime sleepiness, sleep satisfaction and sleep hours at T1.

**Table 6:** Effect of stress on short sleep duration. Multivariate hierarchical logistic regression analysis.

Stress group	Model I^a^		Model II^b^		Model III^c^		Model IV^d^	
	OR	CI95	OR	CI95	OR	CI95	OR	CI95
High stress	52.77***	11.75–236.97	61.16***	13.15–284.43	10.15*	1.44–71.33	12.09*	1.32–111.11
Intermediate high	7.06***	2.74–18.16	8.26***	3.08–22.19	4.22	0.99–17.87	4.78	0.99–22.98
Intermediate low	1.83	0.94–3.54	1.97	0.98–3.95	1.74	0.63–4.82	1.91	0.67–5.45
Low stress	Ref.		Ref.		Ref.		Ref.	

Notes.

*** p < .001,

** p < .01,

* p < .05.

CI95 = 95% confidence interval.

^a^ Crude estimates.

^b^ Adjusted for age, education rank level, geographic origin, marital status, housing, and presence of offspring.

^c^ Adjusted for Model 2 + sleep hours at T1.

^d^ Adjusted for Model 3 + ESS, sleep satisfaction and insomnia symptom score at T1.

**Table 7:** Effect of stress on sleep satisfaction. Multivariate hierarchical logistic regression analysis, according to stress levels.

Stress group	Model I^a^		Model II^b^		Model III^c^		Model IV^d^	
	OR	CI95	OR	CI95	OR	CI95	OR	CI95
High stress	34.95***	11.49–106.34	38.66***	11.80–126.67	6.50*	1.46–28.88	4.90	0.91–26.49
Intermediate high	13.31***	4.22–41.97	13.71***	4.14–45.38	7.34*	1.60–33.76	6.34*	1.23–32.57
Intermediate low	2.43	0.82–7.27	2.04	0.67–6.24	2.18	0.50–9.40	2.08	0.45–9.51
Low stress	Ref.		Ref.		Ref.		Ref.	

Notes.

*** p < .001,

** p < .01,

* p < .05.

CI95 = 95% confidence interval.

^a^ Crude estimates.

^b^ Adjusted for age, education rank level, geographic origin, marital status, housing, and presence of offspring.

^c^ Adjusted for Model 2 + sleep satisfaction at T1.

^d^ Adjusted for Model 3 + ESS, sleep hours and insomnia symptom score at T1.

**Table 8:** Effect of stress on sleepiness. Multivariate hierarchical logistic regression analysis.

Stress group	Model I^a^		Model II^b^		Model III^c^		Model IV^d^	
	OR	CI95	OR	CI95	OR	CI95	OR	CI95
High stress	34.95***	11.49–106.34	54.06***	15.76–185.42	28.18***	4.21–128.45	12.17*	1.22–121.86
Intermediate high	11.69***	3.70–36.94	14.00***	4.11–47.78	19.92**	2.92–135.69	11.08*	1.31–93.35
Intermediate low	3.44*	1.19–9.92	3.17*	1.06–9.48	9.45*	1.51–59.13	10.69*	1.45–78.54
Low stress	Ref.		Ref.		Ref.		Ref.	

Notes.

*** p < .001,

** p < .01,

* p < .05.

CI95 = 95% confidence interval.

^a^ Crude estimates.

^b^ Adjusted for age, education rank level, geographic origin, marital status, housing, and presence of offspring.

^c^ Adjusted for Model 2 + sleepiness score at T1.

^d^ Adjusted for Model 3 + sleep hours, sleep satisfaction and insomnia symptom score at T1.

The crude estimates also indicated that police officers with sleep problems had significantly higher odds of reporting a high level of work-related stress than the reference group (Table 9). Univariate logistic regression analysis showed that insomnia symptoms, sleep hours, sleep satisfaction and daytime sleepiness were significant determinants of distress. Insomnia symptoms (OR = 7.72 CI95% 3.84–15.51) and excessive daytime sleepiness (OR = 10.00 CI95% 5.22–19.16) were associated with high stress levels, while hours of sleep (OR = 0.17, CI95% 0.11–0.28) and sleep satisfaction (OR = 0.14, CI95% 0.08–0.22) had a reverse association. These associations remained significant after correction for demographic variables. However, the addition of sleep complaints that were present at baseline into the model reduced the association between sleepiness and stress. In the fully corrected model, only sleep duration, insomnia symptoms and sleep satisfaction were significant predictors of the perception of occupational stress.

**Table 9:** Effect of sleep problems on distress. Multivariate hierarchical logistic regression analysis.

Sleep at T2	Model I^a^		Model II^b^		Model III^c^	
	OR	CI95	OR	CI95	OR	CI95
DS-SI-EA symptoms	7.72***	3.84–15.51	8.57***	4.02–18.28	3.92***	1.51–10.17
SH-sleep hours	0.17***	0.11–0.28	0.14***	0.08–0.24	0.30***	0.13–0.68
SS-sleep satisfaction	0.14***	0.08–0.22	0.12***	0.07–0.21	0.26***	0.11–0.60
EDS-sleepiness	10.00***	5.22–19.16	13.16***	6.41–28.89	7.84***	2.32–26.42

Notes.

*** p < .001,

** p < .01,

* p < .05.

CI95 = 95% confidence interval.

ST, with/without insomnia; SS satisfied/dissatisfied; SH sufficient/ insufficient sleep loss; EDS normal/excessive daytime sleepiness

^a^ Crude estimates.

^b^ Adjusted for age, education rank level, geographic origin, marital status, housing, and presence of offspring.

^c^ Adjusted for Model 2 + sleep variables at T1.

### Mediation analysis

The results of the mediation analysis are illustrated in Fig 1. The total (unadjusted) effect of occupational stress on MetS was statistically significant (regression coefficient: 0.382, *p* = 0.039) and this was also true for the links between occupational stress and insomnia (“a” pathway, regression coefficient: 0.92, *p*<0.001) and between insomnia and metabolic syndrome (“b” pathway, regression coefficient: 0.452, *p*<0.001). A multiple logistic regression model that took MetS as the dependent variable and both occupational stress and insomnia (i.e. the mean value of insomnia scores at baseline and at the end of follow-up) as independent variables, demonstrated that the relationship between occupational stress and MetS was no longer significant (regression coefficient: -0.066, *p* = 0.78) after data adjustment for insomnia. An assessment of the bootstrapping indirect effect of occupational stress (i.e. mediated by insomnia) revealed that the regression coefficient of such an effect was 0.438, with a bootstrapping confidence interval that did not include zero (i.e. it was statistically significant) ranging from 0.182 to 0.801.

## Discussion and conclusions

This study demonstrates that low sleep quantity and quality are powerful predictors of MetS, and lends support to the hypothesis that the association between some sleep complaints (difficulty sleeping, sleep interruptions, and early awakenings) and MetS, that has been frequently observed in cross-sectional studies, also implies the existence of a causal link. Moreover, our study supports the idea that sleep loss is a causal factor of MetS, and confirms the findings of some previous studies on this topic.

Likewise, the present study demonstrates that sleep problems mediate the effect of occupational stress on MetS. Should this result be generalized in longitudinal studies on other populations, researchers would have at their disposal an indicator of MetS risk that would be much simpler to use than the questionnaires for occupational stress. In fact, the latter require a certain competence on the part of the physician who must first of all select the stress model best suited to the population and then evaluate the results correctly. Moreover, workers may find it difficult to express their distress openly. Although investigating sleep symptoms can be much easier than investigating stress, very few doctors systematically collect sleep-related symptoms.

The study also sheds new light on the relationship between stress and sleep problems. Relatively consistent findings in the literature have demonstrated that experiencing job stress enhances the risk of sleep problems. The results of the present study clearly show a bidirectional relationship between sleep and stress: workers exposed to chronic occupational stress have an increased incidence of sleep problems, and bad sleepers suffer more from occupational stress factors than good sleepers. This result reinforces similar observations from previous studies. Data from the Swedish Longitudinal Occupational Survey of Health (SLOSH) detected reverse and reciprocal relationships in addition to the commonly hypothesized causal relationships between occupational stress and sleep problems [73]. More recently, a Norwegian study confirmed the commonly hypothesized unidirectional forward associations between occupational stress and sleep problems among women, and demonstrated a reverse and reciprocal relation between work stressors and sleep problems among men [74]. Data from the Workplace Bullying and Harassment cohort in Denmark also found evidence for a reciprocal association [75]. A small reverse effect, with reduced sleep duration (but not sleep quality) predicting increased post-traumatic stress, was also observed in World Trade Center (WTC) responders [76].

The results of this research should be interpreted principally in relation to members of the police force. Poor sleep quality is very common in the law enforcement profession. According to a recent systematic review with meta-analysis, 51% of police officers report bad sleep quality [77]. Sleep problems have been associated with night and evening work schedules [78] or abnormal work hours [79]. However, very few studies have been conducted that take into consideration work-related stressors other than night shifts, or the association between stress and sleep problems. Some studies have examined the way stress can alter sleep quality, depending upon the different coping strategies adopted by police officers [80]. Reverse casualty, i.e. that sleep problems can influence the response to stress, has also been studied: a small sample of Iowa law enforcement officers with short sleep duration or poor sleep quality reported more stress, burnout, and depression symptoms than controls [81]. The present study confirms previous experiences, thus indicating that sleep problems in police officers may be connected with MetS and cardiovascular risk [82, 83].

The results of this study on policemen confirm findings reported in research conducted on the general population and other categories of workers as regards the association between stress and cardiovascular risk factors. Studies undertaken in the 1960s and conducted up to the present day have revealed that work-related stress is associated with cardiovascular risk [84]. A summary of the studies that used the ERI model to assess occupational stress as the imbalance between the effort required for work tasks and the rewards received, specifically indicates that stress increases cardiovascular risk [85]. Furthermore, a very recent systematic review of the literature reported a strong association between stress and alteration in biological indicators that is especially consistent with MetS, alteration in blood lipids and variability in heart rate [86]. Similar evidence has been found with the DCS model [87]. Surveys conducted on over 600,000 workers confirm that stress induces MetS [88–90]. Our study carries forward this research by indicating that sleep acts as a mediating factor in the relationship between occupational stress and cardiovascular risk.

The main strength of the present study lies in the fact that it benefited from a selected cohort in which exposure to relevant risk factors (stress, abnormal shifts) was homogeneous and did not include any important confounders for MetS (gender, diet, physical activity). A further strong point was the frequent assessment of stress levels by means of two complementary and standardized questionnaires thus enabling the police officers to be classified into different levels of resilience.

The results of this study need to receive experimental confirmation in other working populations. The main limitation of the study was the scant number of observations that inevitably led to a high standard error. Our findings must be interpreted with caution, in consideration of the range of the confidence intervals that makes it difficult to relate our observations to the general population. The observed sample can however be considered similar to other police units engaged in maintaining law and order. The limitation due to the meager number of workers was offset by constant participation and the negligible loss of cases. The cheap and simple method used in this research should encourage occupational health services to verify our results by conducting similar studies in other productive sectors.

The conclusions of a previous study [18] highlighted the need to avoid stress in police officers in order to prevent MetS, while the present study indicates the importance of avoiding a vicious circle involving stress and sleep problems as this would be more likely to cause MetS than stress alone. Health promotion in the police force should have the two-fold aim of increasing resilience to stressors and spreading healthy sleep patterns and proper sleep hygiene practices.
